# Biochemical, genomic and structural characteristics of the Acr3 pump in *Exiguobacterium* strains isolated from arsenic-rich Salar de Huasco sediments

**DOI:** 10.3389/fmicb.2022.1047283

**Published:** 2022-11-03

**Authors:** Juan Castro-Severyn, Coral Pardo-Esté, Ingrid Araya-Durán, Valentina Gariazzo, Carolina Cabezas, Jorge Valdés, Francisco Remonsellez, Claudia P. Saavedra

**Affiliations:** ^1^Laboratorio de Microbiología Aplicada y Extremófilos, Departamento de Ingeniería Química, Universidad Católica del Norte, Antofagasta, Chile; ^2^Laboratorio de Microbiología Molecular, Facultad de Ciencias de la Vida, Universidad Andres Bello, Santiago, Chile; ^3^Center for Bioinformatics and Integrative Biology, Facultad de Ciencias de la Vida, Universidad Andres Bello, Santiago, Chile; ^4^Centro de Investigación Tecnológica del Agua en el Desierto (CEITSAZA), Universidad Católica del Norte, Antofagasta, Chile

**Keywords:** arsenic, ACR3, efflux pumps, resistance, *Exiguobacterium*

## Abstract

Arsenic is a highly toxic metalloid of major concern for public safety. However, microorganisms have several resistance mechanisms, particularly the expression of arsenic pumps is a critical component for bacterial ability to expel it and decrease intracellular toxicity. In this study, we aimed to characterize the biochemical, structural, and genomic characteristics of the Acr3 pump among a group of *Exiguobacterium* strains isolated from different sites of the arsenic-rich Salar de Huasco (SH) ecosystem. We also determined whether the differences in As(III) resistance levels presented by the strains could be attributed to changes in the sequence or structure of this protein. In this context, we found that based on *acr3* sequences the strains isolated from the SH grouped together phylogenetically, even though clustering based on gene sequence identity did not reflect the strain’s geographical origin. Furthermore, we determined the genetic context of the *acr3* sequences and found that there are two versions of the organization of *acr3* gene clusters, that do not reflect the strain’s origin nor arsenic resistance level. We also contribute to the knowledge regarding structure of the Acr3 protein and its possible implications on the functionality of the pump, finding that although important and conserved components of this family of proteins are present, there are several changes in the amino acidic sequences that may affect the interactions among amino acids in the 3D model, which in fact are evidenced as changes in the structure and residues contacts. Finally, we demonstrated through heterologous expression that the *Exiguobacterium* Acr3 pump does indeed improve the organisms As resistance level, as evidenced in the complemented *E. coli* strains. The understanding of arsenic detoxification processes in prokaryotes has vast biotechnological potential and it can also provide a lot of information to understand the processes of evolutionary adaptation.

## Introduction

Arsenic (As) is one of the most ubiquitous, non-essential, and highly toxic metalloids in the world; therefore, resistance mechanisms to its presence evolved very early and exist in all domains of life (Mateos et al., 2017). The release of arsenic from minerals into sediments and water can occur because of natural phenomena such as volcanic activities and erosion, but most arsenic pollution is due to human activities (Hoang et al., 2010; Zhu et al., 2014), such as mining and agriculture (Islam et al., 2004; Norra et al., 2005).

The inorganic forms arsenite [As(III)] and arsenate [As(V)] are more prevalent than the organic forms of arsenic in terrestrial environments, and these species can act as selective pressures on the ecosystems, especially for microorganisms. In bacteria, arsenic resistance *ars* genes are widespread and commonly found as arsenic resistant operons in two different configurations: consisting of three (*arsRBC*) or five (*arsRDABC*) genes, mostly organized into single transcriptional units (Mateos et al., 2017). Also, our research group found that an accessory arsenic efflux pump gene (*acr3*) is found outside the *ars* operon (Castro-Severyn et al., 2017), which also has been described for other species (Andres and Bertin, 2016).

The simplest *ars* operons have an As(III)-responsive trans-acting transcriptional regulator (ArsR), an arsenate reductase (ArsC), and the arsenite efflux pump (ArsB) (San Francisco et al., 1989, 1990; Wysocki et al., 1997; Ghosh et al., 1999; Meng et al., 2004). More complex forms appear to be later adaptations to enhance the activity of existing pumps, including an ATPase (ArsA) stimulated by As(III) that may interact with ArsB or Acr3 (Yang et al., 2012) and the metallochaperone/trans-acting repressor (ArsD). Other arsenic resistance genes have been characterized more recently, such as ArsM, ArsI, ArsP, and ArsH, some of which improve resistance to organic arsenicals (Ji and Silver, 1992; Gladysheva et al., 1994; Lin et al., 2006; Yang et al., 2012; Li et al., 2016; Serrato-Gamiño et al., 2018).

However, arsenic efflux is the most important arsenic detoxification mechanism; in this context, six types of arsenic efflux transporters have been characterized so far (ArsB, Acr3, ArsJ, ArsP, ArsK, and MSF1; Achour et al., 2007; Garbinski et al., 2019). The relative abundance of *arsB* and *acr3* in microorganisms is very high, even in low arsenic-contaminated soils, and they show a significant correlation with arsenic concentrations (Wang et al., 2020; Castro-Severyn et al., 2021), providing an interesting biomarker for arsenic contamination (Cai et al., 2009; Poirel et al., 2013).

Even though the transporters ArcB and Acr3 share the same function, they evolved independently and show no sequence similarities (Mansour et al., 2007; Yang et al., 2015; Garbinski et al., 2019). ArsB is classified within the ion transporters superfamily and is found only in prokaryotes, while Acr3 proteins are smaller and belong to the bile/arsenite/riboflavin transporter (BART) superfamily and are found widespread in bacteria, archaea, fungi, and some plants (Mansour et al., 2007; Castillo and Saier, 2010; Yang et al., 2015). Shi et al. (2018) constructed a Neighbor-joining phylogenetic tree of arsenic efflux proteins and found that there was a clear association of proteins by function, as ArsB and Acr3 sequences formed distinct groups together, as did ArsK, ArsJ, and MFS1 sequences, but they were clearly divergent from each other at the subgroup level. ArsB and Acr3 are dominant in abundance compared to other As-pumps, but they are also the most ancient as it has been proposed that the minor types of pumps have a complementary function (Yang et al., 2015).

Resistance to As(III) by extrusion mediated by the Acr3 transporter is the most common tolerance mechanism found in prokaryotes and eukaryotes (Maciaszczyk-Dziubinska et al., 2012; Markowska et al., 2015; Yang et al., 2015). In yeast (*Saccharomyces cerevisiae*), arsenic resistance requires at least three genes: *ACR1*, *ACR2*, and *ACR3,* of which Acr3p is an oxyanion transporter that extrudes As(III) and is positively regulated by Acr1p (Wysocki et al., 1997). The impact of gene dose or multicopy on the ability to resist arsenic has also been previously addressed with variable results (Mateos et al., 2017).

The heterologous expression of Acr3 from yeast or plants in agronomical important species that are susceptible to arsenic is of a great biotechnological interest to generate safer and more resistant crops. Previous research has achieved increased arsenic tolerance, through enhanced arsenic efflux, in *Escherichia coli* AW3110 (Bandyopadhyay and Das, 2016), yeast (Bobrowicz et al., 1997; Wysocki et al., 1997; Ghosh et al., 1999), as well as *Oryza sativa* (rice), *Arabidopsis thaliana,* and *Nicotiana tabacum* plants (Ali et al., 2012; Duan et al., 2012; Chen et al., 2017).

Our research group has studied different strains of the *Exiguobacterium* bacterial genus isolated from the Chilean Altiplano and their genomes, focusing on their arsenic-resistance traits. These bacteria can thrive under several stressors, including high concentrations of arsenic in the sediments of Salar de Huasco (SH). This area is known for its high microbial biodiversity, as well as the complexity of different niches in a relatively small space, including salinity and arsenic gradients (Castro-Severyn et al., 2020). At metagenomic level, the most abundant genes related to arsenic in these bacterial communities of the SH are *acr3* followed by *arsB* and *arsJ*, indicating that the As(V) reduction and subsequent As(III) expulsion mechanism is the most prevalent detoxification means used by bacteria in this ecosystem (Castro-Severyn et al., 2021).

**Figure 1:**
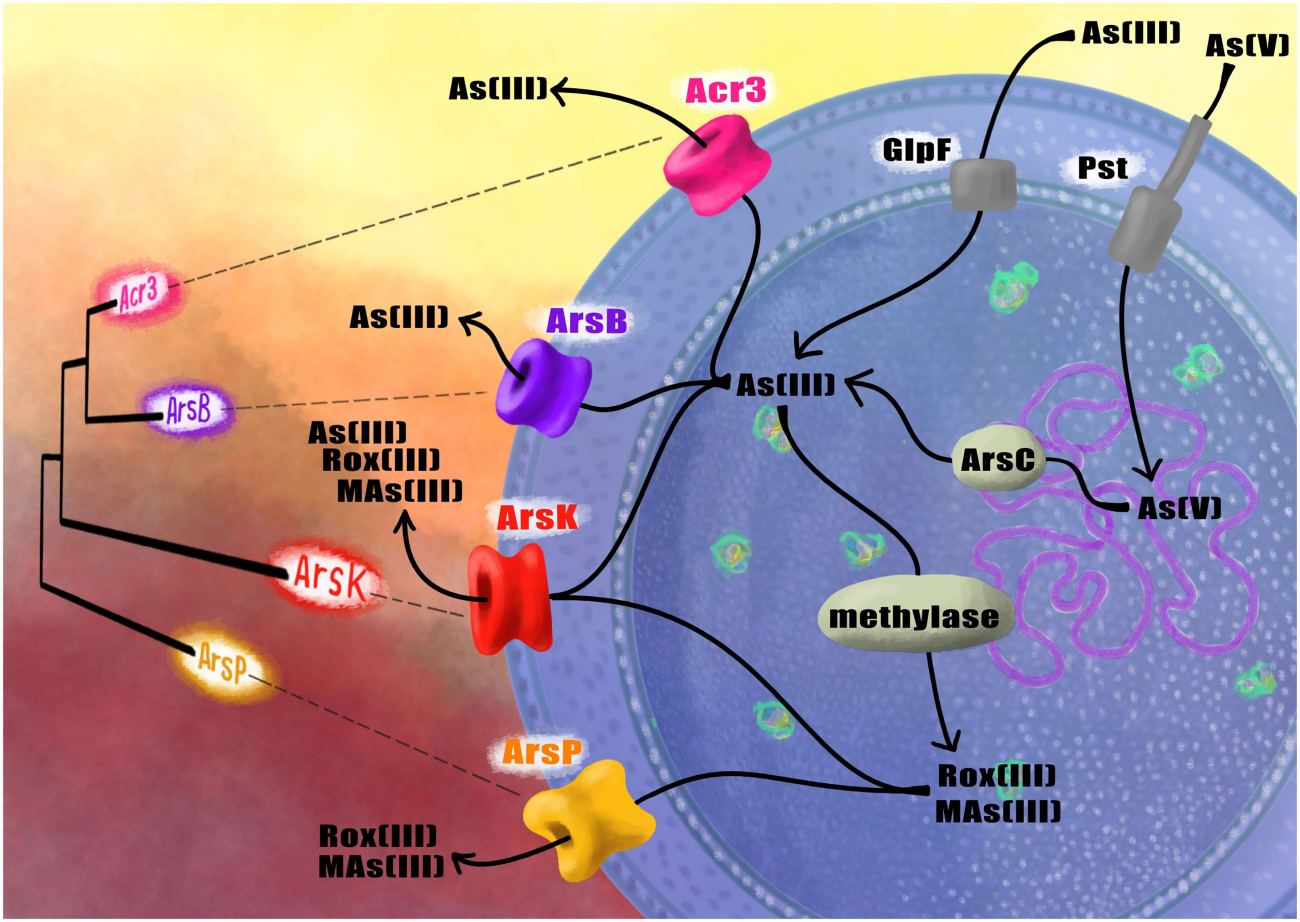
Schematic representation of the phylogenetic relationships between the arsenic efflux pumps found in *Exiguobacterium* and their function inside the cell to face arsenic.

Furthermore, we have studied the genome and physiology of the *Exiguobacterium* sp. SH31 strain that is able to grow in up to 10 mM arsenite and 100 mM arsenate, and we found that the expression of the *ars* operon and *acr3* was strongly-induced in response to both toxics, suggesting that the arsenic efflux pump Acr3 mediates arsenic resistance in *Exiguobacterium* sp. SH31 (Castro-Severyn et al., 2017). Furthermore, the *acr3* gene is not frequent among this bacterial genus; it was only found in strains S17 and SH31, both isolated from the Altiplano. Their protein sequences share 94% identity and the conservation of critical residues between both proteins, which could partially explain the high arsenic resistance of these strains (Ordoñez et al., 2015; Castro-Severyn et al., 2017). Interestingly, the *Exiguobacterium* strains isolated from SH possess four different arsenic pumps: ArsB, ArsK, ArsP and Acr3 (Figure 1; Castro-Severyn et al., 2020).

Moreover, our group has reported the differential expression of the pumps present in three *Exiguobacterium* strains (with different levels of arsenic resistance) at both transcriptional (Castro-Severyn et al., 2020) and proteomic (Castro-Severyn et al., 2019) levels induced by arsenic. In this study, we characterized the biochemical, structural, and genomic characteristics of the Acr3 pump among a group of *Exiguobacterium* strains isolated from different sites of the arsenic-rich SH ecosystem. In order to address whether possible changes in the protein sequence and structure among the strains could be the source of the displayed differences in arsenic resistance levels presented by the strains, something that has not been done previously nor reports are available.

## Materials and methods

### Similarities between the *acr3* gene sequences

To make comparative analyses at the gene level, we extracted the *acr3* sequences from the genomes of the 10 *Exiguobacterium* strains isolated from the SH, including the SH31 strain (Castro-Severyn et al., 2020), in addition to some reference sequences as shown in Table 1. A phylogeny was constructed to evaluate the evolutionary relationships between the *acr3* genes. For this, the gene sequences were codon-aligned with RevTrans v2.0 (Rasmus and Pedersen, 2003), the alignment was used to infer phylogeny (GTR model) with FastTree v2.1.10 (Price et al., 2009) and FigTree v1.4.4 (Rambaut, 2009) was used to visualize the tree. Also, MAFFT v7 (Katoh and Standley, 2013) and PAL2NAL v14 (Suyama et al., 2006) tools were used to align the protein sequences, converting the multiple sequence alignment to the corresponding DNA codon alignment, which was used to calculate synonymous (dS) and non-synonymous (dN) substitution rates. Moreover, to analyze the degree of similarity and clustering between the different sequences, an identity matrix was calculated using pyANI v0.2.10 (Pritchard et al., 2016) using a multi-dimensional scaling (MDS) ordination with the R environment v4.0.3 (base and the packages stats v4.0.3, ggplot2 v3.3.5 and dplyr v1.0.7). Another phylogeny was constructed with the Acr3 amino acidic sequences, using MAFFT v7 (Katoh and Standley, 2013) with an L-INS-i strategy for iterative refinements, and a maximum-likelihood phylogeny tree was generated with IQ-TREE (Nguyen et al., 2015; 10,000 bootstrap) and visualized with FigTree v1.4.4 (Rambaut, 2009). The *acr3* genomic context of the strains was visualized with Geneious v2022.1.1 (Kearse et al., 2012) and the homology of neighboring orthologous genes was evaluated using Blast (Altschul et al., 1990).

**Table 1:** *Exiguobacterium* strains and other bacterial reference Acr3 sequences.

Strains	GenBank accessions		MIC [mM]	
	Genes	Proteins	As(III)	As(V)
*Exiguobacterium* sp. SH0S1	GCA_004337165.1	WP_131507397.1	7.5	200
*Exiguobacterium* sp. SH0S2	GCA_004337185.1	WP_131489940.1	10	200
*Exiguobacterium* sp. SH0S7	GCA_004337195.1	WP_131443547.1	20	150
*Exiguobacterium* sp. SH3S1	GCA_004337105.1	WP_131469704.1	15	200
*Exiguobacterium* sp. SH3S2	GCA_004337285.1	TCI59634.1	2.5	200
*Exiguobacterium* sp. SH3S3	GCA_004337115.1	TCI44176.1	7.5	200
*Exiguobacterium* sp. SH31	GCA_001816105.1	WP_084813073.1	10	100
*Exiguobacterium* sp. SH4S7	GCA_004336795.1	TCI37681.1	10	200
*Exiguobacterium* sp. SH5S4	GCA_004337045.1	WP_131458986.1	15	200
*Exiguobacterium* sp. SH5S13	GCA_004337085.1	WP_131468614.1	15	100
*Exiguobacterium* sp. SH5S32	GCA_004336775.1	TCI46015.1	15	100
**References**				
*Corynebacterium glutamicum* ATCC 13032	Cgl0262	BAB97655.1	12	>500
	Cgl1470	BAB98863.1		
	Cgl1510	BAB98903.1		
*Alkaliphilus metalliredigens* QYMF	Amet_1828	ABR47998.1	–	–
	Amet_0984	ABR47201.1		
	Amet_3996	ABR50078.1		
*Bacillus subtilis* 168	ASM904v1	NP_390456.2	–	–
*Exiguobacterium* sp. S17	GCA_000411915.1	WP_035385877.1	10	150
*Exiguobacterium mexicanum* HUD	GCA_000763125.1	WP_034781870.1	–	–
*Exiguobacterium aurantiacum* PN47	GCA_001766415.1	WP_083274029.1	–	–

### Acr3 protein modeling and structural analysis

Variations in Acr3 amino acid sequences from the eleven SH *Exiguobacterium* strains were discovered by alignment using clustalW (Madeira et al., 2019), and visualized with Jalview v2.11.1.4 (Waterhouse et al., 2009). The topology of Acr3 and transmembrane segments was predicted using TMHMM (Krogh et al., 2001) as well as PSIPRED (Jones, 1999) which predicts transmembrane helices, extracellular and cytoplasmic topologies. Moreover, all three-dimensional models of Acr3 were predicted using the ColabFold server (Mirdita et al., 2022), which uses the AlphaFold v2.0 (Jumper et al., 2021; Varadi et al., 2022) program, and the MMseqs2 (Steinegger and Söding, 2017) software for the generation of multiple sequence alignments and for template searching in order to construct the protein structure. Additionally, the predicted structures were relaxed to improve the geometry of the side chains by removing stereochemical violations and clashes. The relaxation of the structures was performed by an iterative restrained energy minimization procedure, with additional harmonic restraints that keep the system near its input structure. The minimization procedure was performed using the OpenMM v7.3.1 (Eastman et al., 2017) simulation package with the Amber99sb force field (Hornak et al., 2006), considering a tolerance of 2.39 kcal/mol and an unbounded maximum number of steps, which are OpenMM’s default values. The templates used for the AlphaFold2 predictions were the bile acid sodium transporters from *Neisseria meningitidis* and *Yersinia frederiksenii* bacteria (PDBs: 3ZUX, 3ZUY, 4N7W). Also, the per-residue confidence metric pLDDT value of AlphaFold was used to estimate how well the prediction of Acr3 proteins was performed, whilst the VMD program (Humphrey et al., 1996) was used to visualize the protein models, thus generating the structural superpositions through the Stamp Structural Alignment feature of MultiSeq (Roberts et al., 2006) and to measure the intra hydrogen bonds of proteins by the Hbonds plugin and default parameters. Furthermore, a contact map for the protein structures was created using CMView (Vehlow et al., 2011), selecting as contact type the alpha carbons of the protein, a cutoff distance of 8.0 angstroms and the Needleman-Wunsch sequence alignment. In order to understand the energy changes associated with the amino acids that vary between the Acr3 proteins and their contact network, the binding energy of each amino acid under study was calculated by the MM-GBSA method (Gohlke et al., 2003; Gohlke and Case, 2004) as follows:


$${\mathrm{\Delta G}}_{\text{bind}}=G_{\text{TOTAL}}\left(\mathrm{AB}\right)-G_{\text{TOTAL}}\left(A\right)-G_{\text{TOTAL}}\left(B\right)$$

where AB corresponds to the contact network formed by the amino acid that varies (for example, residue 10) and the residues in its environment that are at a distance of 5 angstrom. The term A corresponds only to the amino acid that varies, and B corresponds only to the residues of the environment without considering A. G_TOTAL_ corresponds to the sum of HMM and G_solv_. HMM is the enthalpy, which corresponds to the molecular mechanics energies (bonds, angles, VDW, and electrostatic contribution). G_solv_ represents the solvation free energy obtained by the Generalized Born approach and solvent accessible surface area (SASA).

### Strains and culture conditions

The strains used in this study are listed in Table 2. *Escherichia coli* strains were grown routinely at 37°C in Luria Bertani (LB) medium with shaking (150 rpm). When required, LB was supplemented with ampicillin (Amp, 100 μg/ml), Tetracycline (Tet, 10 μg/ml) or Chloramphenicol (Cam, 25 μg/ml). The *Exiguobacterium* sp. SH31 strain was grown in LB medium with shaking (150 rpm) at 25°C.

**Table 2:** Bacterial strains used in this study.

Strain	Relevant characteristics	Source (Reference)
*Exiguobacterium* sp. SH31	WT, arsenic resistant *arsRDCBA* + *acr3 +*	Our group (Castro-Severyn et al., 2017)
*E. coli* BW25113	*E. coli* K-12 derivative *arsRCB* + *acr3 -*	CGSC* (Baba et al., 2006)
*E. coli* AW3110	*E. coli* K-12 derivative Δ*ars*::Cam *arsRCB - acr3 -*	CGSC* (Carlin et al., 1995)
*E. coli* BW25113/pBR322-*acr3*	BW25113 strain transformed with a derivative pBR322 vector carrying the *Exiguobacterium* sp. SH31 *acr3* gene	This work
*E. coli* AW3110/pBR322-*acr3*	AW3110 strain transformed with a derivative pBR322 vector carrying the *Exiguobacterium* sp. SH31 *acr3* gene	This work

* The *E. coli* Genetic Stock Center (https://cgsc.biology.yale.edu/index.php).

### Genetic complementation with *acr3*

To complement the BW25113 and AW3110 strains, the *acr3* gene from *Exiguobacterium* sp. SH31 was amplified by PCR using the primers (F: 5′- CCCAAGCTTTTGAGTCGATTCGAGAAATT-3′ and R: 5′- CCCGGATCCCTAACCGAGTCGACGTTC-3′) which introduces the HindIII and BamHI restriction sites (underlined), respectively, using gDNA as a template. The PCR product and the pBR322 vector (Invitrogen) were then digested with both enzymes and the digested product was cloned between the vector HindIII – BamHI sites, interrupting the *tet* gene (losing the resistance). The generated pBR322-*acr3* vector was transformed by electroporation into competent BW25113 and AW3110 cells. Correct transformants were selected by plating cells in LB medium (agar 10 gr/L) with the appropriate antibiotics.

### Arsenic minimal inhibitory concentration

Minimal inhibitory concentration (MIC) assays for As(III) and As(V) were performed for all strains. Briefly, bacterial cultures in LB broth (supplemented with corresponding antibiotics) were grown at 37°C with constant agitation (150 rpm) until OD_600_ = 0.4. Subsequently, we set up a microplate with dilutions of As(III): NaAsO_2_ and As(V): Na_3_AsO_4_ to final concentrations ranging from 0.1 to 25 mM and from 1 to 200 mM, respectively. Controls were made by adding fresh medium to the corresponding well instead of arsenic. Each well was inoculated with the culture in 1:20 ratio in LB medium. Finally, the plates were incubated at 37°C for 12 h with constant agitation, and OD_600_ values were read with an Infinite 200 PRO Microplate Reader (Tecan).

### Growth monitoring

The growth of all strains was monitored in LB media with different As(III) (0.5, 1, 1.5 and 2.5 mM) and As(V) (5, 10, 20, 50 mM) concentrations and the corresponding antibiotics. Cells were incubated at 37°C with constant agitation at 150 rpm for 24 h. The OD_600_ was measured every hour using an Infinite 200 PRO Microplate Reader (Tecan). Curves were plotted using the R package ggplot2 (Wickham, 2016).

### Colony-forming unit determination

The number of viable microorganisms in control and arsenic conditions was estimated by the method described by Miles et al. (1938) and was expressed as CFU/ml. Briefly, cultures from all the strains were grown until their exponential phase (OD_600_ ~ 0.4). For the BW25113 strains the As(III) and As(V) concentrations were 1 and 10 mM respectively, while for the AW3110 strains, the corresponding concentrations were 0.5 and 5 mM. The cultures were then serially diluted, plated into LB medium and incubated for 12 h prior to the colony counting process. Plots were made using the R package ggplot2 (Wickham, 2016).

## Results

### Relationships among the *acr3* gene sequences

**Figure 2:**
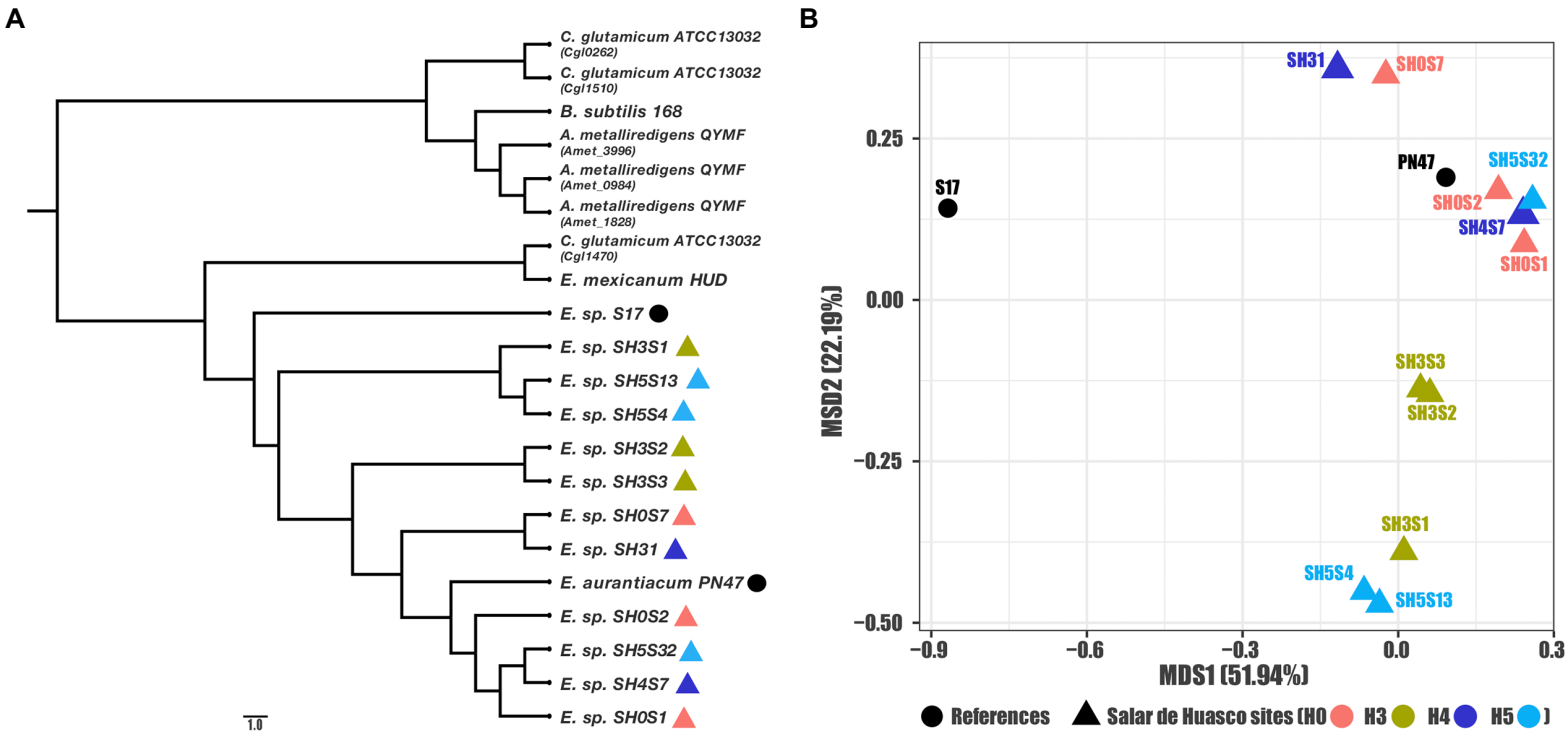
Genomic relationships among the studied *acr3* sequences. **(A)** Phylogenetic reconstruction for all compared gene sequences (*Exiguobacterium* strains and references) and **(B)** MDS ordination using sequence identities as distance (for the *Exiguobacterium* strains and close related references).

We characterized at a genomic and structural level these sequences and their attributes, to identify differences among the data set. First, to evaluate the evolutive associations between known *acr3* genes, we determined the phylogenetic relationships between the nucleotide sequences belonging to *Exiguobacterium* strains isolated from the SH; for comparisons we also included references of *acr3* sequences belonging to other characterized *Exiguobacterium* strains as well as other bacterial species. Our results show that all *Exiguobacterium* strains are monophyletic and closely clustered together, belong to a different group compared to the other genera. In addition, the SH strains are grouped together, being the S17 strains the closest, which comes from a similar environment, also pressured by arsenic (Figure 2A). We also evaluated the mode and strength of selection pressures in the *acr3* sequences by analyzing codon evolution and temporal dynamics through the calculations of dN/dS ratios, using the *acr3* sequence from the S17 strain as reference. The S17 is a closer but external strain, providing proper resolution to evaluate relationships between SH strains sequences (Supplementary Table S1). We found that all the studied *acr3* sequences are under negative selection (with similar dN/dS ratios ranging from 0.1202 to 0.1403) diverging from S17 sequence which seems to be from an independent branch. This is also reflected by the sequence identity values, which ranged from 92.5 to 94.4%. From the amino acidic point of view the phylogeny analysis broadly reflects the distribution observed at genomic level, separating the sequences into three main groups (Supplementary Figure S1), where the closest relationships are maintained.

Moreover, as these sequences are phylogenetically close to each other and to further characterize the relationships between the *acr3* sequences in genomes isolated from SH, we calculated the identity matrix and used them as distances to determine relationships or clustering between the evaluated strains by MSD ordination. Our results show a clear pattern of association where the sequence from the S17 strain isolated from the Argentinian Altiplano is the outliner as expected, and another four clusters are formed within the sequences from the *Exiguobacterium* strains isolated from SH (Figure 2B). Furthermore, we determined that the associations between sequences are not correlated with the specific geographic site, as we found that one group is formed by sequences from strains isolated from site H5 and H3, another includes only strains from H3, also a third cluster includes sequences from sites H4 and H0 and the biggest group is formed by sequences associated with H0, H4, and H5 sites as well as the PN47 reference. Likewise, the clustering observed in the ordination plot corresponds to associations found on the phylogeny tree.

**Figure 3:**
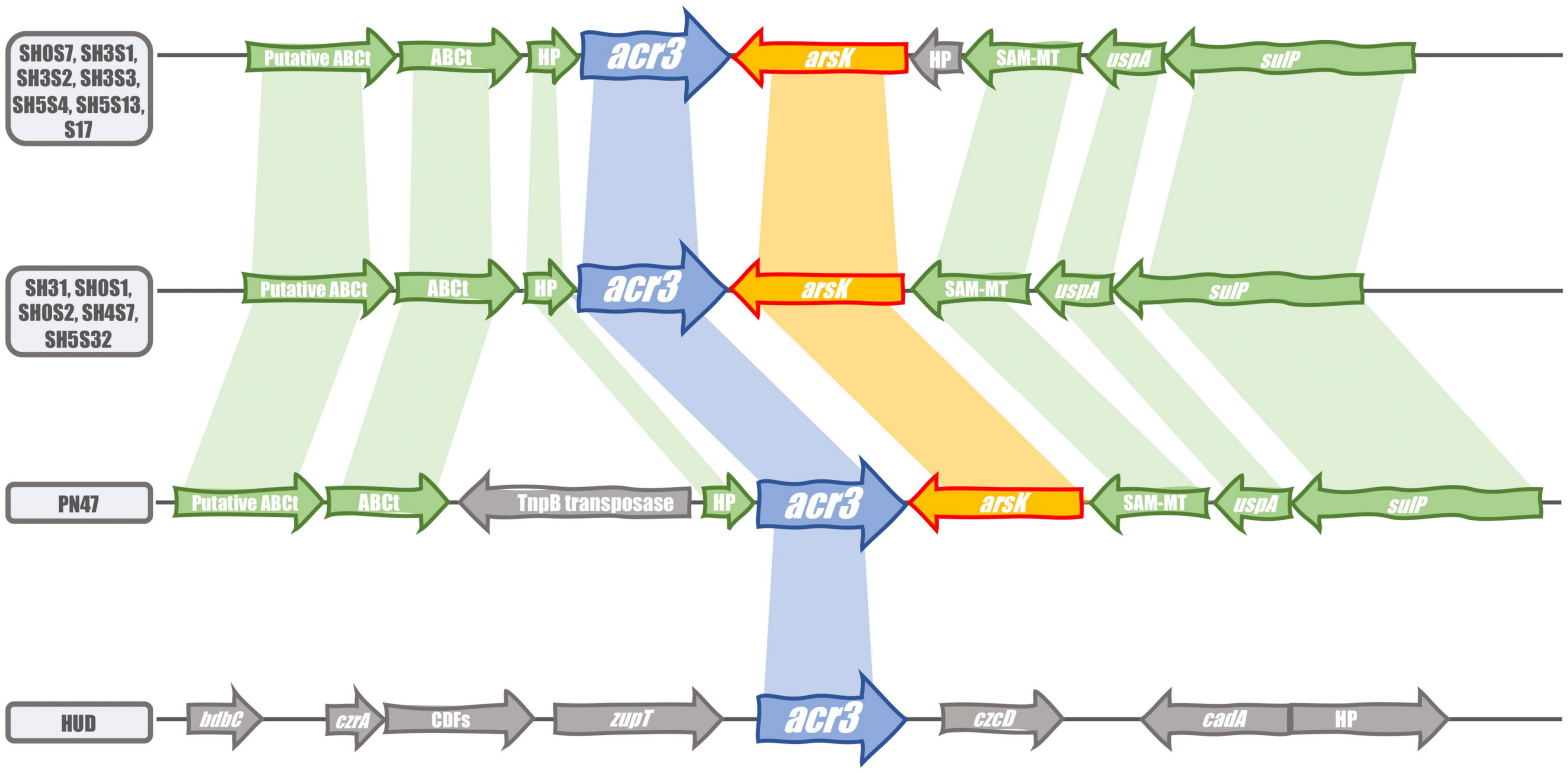
Comparison of the *acr3* gene cluster organization in the compared strains. Blue and orange arrows display the *acr3* and *arsK* genes respectively; green arrows show the conserved flanking genes in the *acr3* gene context and gray arrows display the uncommon genes. Shaded color connections represent gene conservation.

The genomic context of the *acr3* gene is organized in two versions in the studied strains (Figure 3). The first corresponds to 9 genes - composed of two ABC transporters, two hypothetical proteins, the universal stress protein *uspA*, the sulfate transporter *sulP*, a SAM-dependent methyltransferase and the two arsenic transporters *arsK* and *acr3* - in strains SH0S7, SH3S1, SH3S2, SH3S3, SH5S4, SH5S13 and the reference S17. The second version corresponds to 8 of the same genes with the same organization, with the absence of one of the two hypothetical proteins. Such a context was found in the SH31, SH0S1, SH0S2, SH4S7 and SH5S32 strains. The missing hypothetical protein does not have a known function but seems to belong to the DnaJ/Hsp40 cysteine-rich domain superfamily (SSF57938). The reference strain *E. aurantiacum* PN47 also has this second gene context organization, with the addition of a transposase related protein (TnpB), which could have been integrated afterwards. However, *acr3* in *E. mexicanum* HUD strain is in a totally different context with genes related to metal resistance and transport with one hypothetical protein, one cation diffusion facilitator (CDF) family protein, *bdbC* (thiol-disulfide oxidoreductase), *czrA* (zinc, cobalt, nickel and cadmium transcriptional repressor), *zupT* (zinc transporter), *czcD* (Metal cation efflux), and *cadA* (Cadmium-transporting ATPase) genes. These organizations are mostly reflected in the phylogeny and ordination analyses. Consistently, the Acr3 from the HUD strain is phylogenetically closer to those of *C. glutamicum* ATCC13032, being outside the *Exiguobacterium* clade, which could be a hint of HGT in this case.

### Molecular modeling of the Acr3 protein

**Figure 4:**
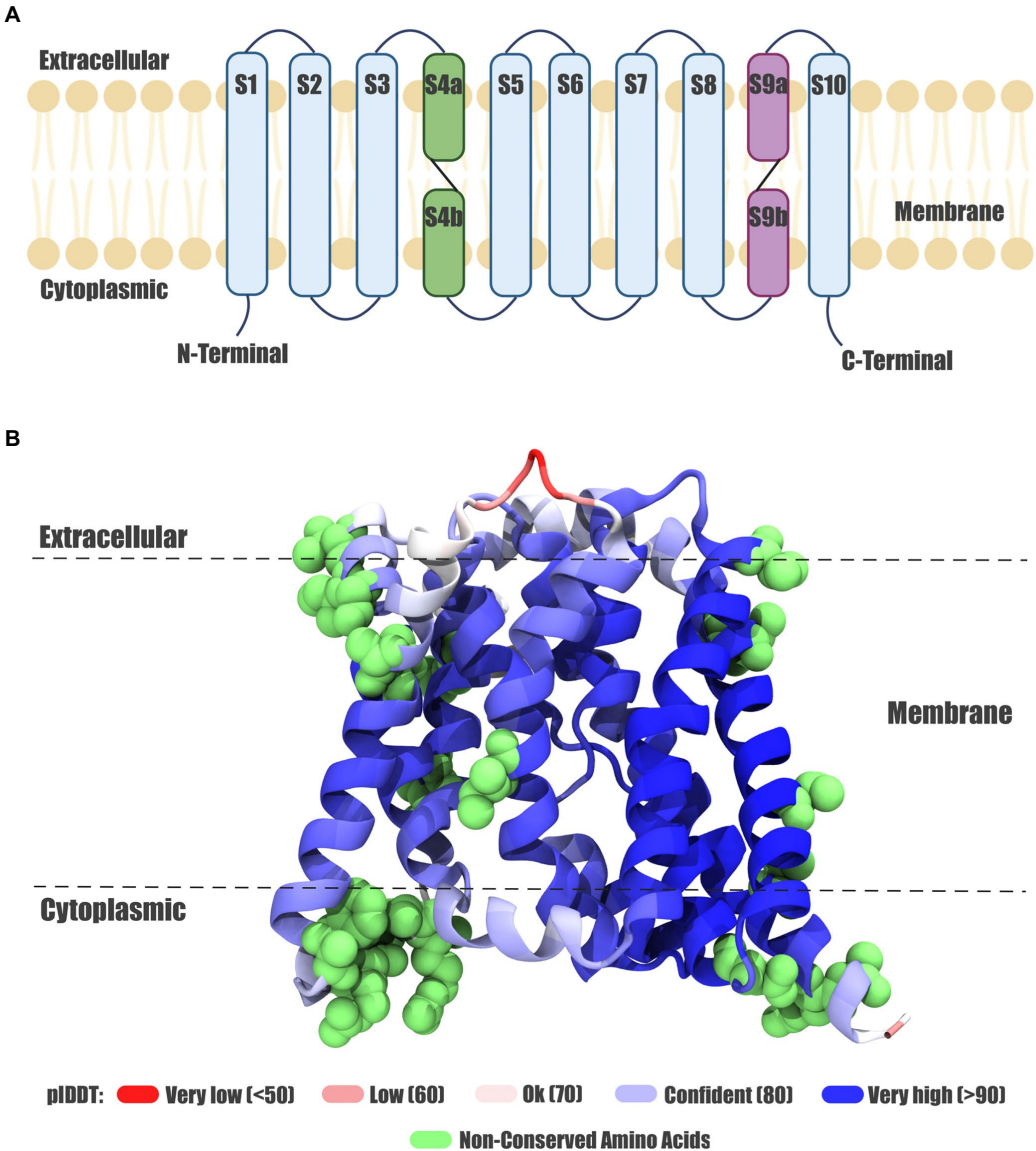
*Exiguobacterium* Acr3 protein model. **(A)** Topology of the Acr3 protein. The ten transmembrane helices are shown, the eight continuous helices in light blue and discontinuous helices in purple and green. **(B)** Acr3 protein model from the SH0S1 strain, showing the three-dimensional conformation and the spatial orientation of the ten transmembrane helices; color corresponds to the modeling confidence level and green spheres represent the non-conserved amino acids.

To determine whether the differences and similarities at the genetic level are reflected in the 3D protein structure we characterize the Acr3 sequences of the *Exiguobacterium* studied strains and other sequences used as references trough molecular structural modeling, thus predict the protein structure and identify key amino acids (Figure 4). The results show that the predicted proteins have ten segments corresponding to transmembrane helices (Figure 4A). The model generated indicates a high confidence at each position, as can be seen in the *E*. sp. SH0S1 Acr3 protein model (Figure 4B). Moreover, these Acr3 protein models from *Exiguobacterium* and other species were structurally superimposed by using the crystal structure of the bile acid sodium transporter from *Neisseria meningitidis* (PDB: 3ZUX_A) as a template, which shares 23% identity (sd = 0.006) and an E-value of 9E-4 (sd = 0.0009) compared to the Acr3 modeled proteins. The RMSD (Root Mean Square Deviation) distance measurement between alpha carbons was calculated for all proteins against the template (Supplementary Table S2).

Based on the predicted structure, the main transmembrane helix of *Exiguobacterium* proteins appears to have similar structural features and orientations, with small differences compared to the reference proteins (as can be seen in Supplementary Figure S2A). However, in the reference structures belonging to *Bacillus subtilis* 168, *Corynebacterium glutamicum* ATCC 13032 (BAB98903.1), *Corynebacterium glutamicum* ATCC 13032 (BAB97655.1), *Alkaliphilus metalliredigens* QYMF (ABR47998.1), *Alkaliphilus metalliredigens* QYMF (ABR47201.1) and *Alkaliphilus metalliredigens* QYMF (ABR50078.1) a beta sheet in the extracellular/transmembrane region interface (S2 segment) is observed, which is missing in the Acr3 proteins from *Exiguobacterium* strains. Also, when analyzing and comparing the structures of Acr3 proteins from *Exiguobacterium* strains, we found a clear diversification in three clusters (Supplementary Figure S2B). The first one (represented in orange) is formed by *E.* sp. SH0S1, the second one (in green) is comprised of *E*. sp. SH0S2, *E*. sp. SH0S7, *E*. sp. SH4S7 and *E*. sp. SH5S32, whilst the third one (in grey) is formed by *E*. sp. SH3S1, *E*. sp. SH3S2, *E*. sp. SH3S3, *E*. sp. SH5S4, *E*. sp. SH5S13 and *E*. sp. SH31. However, this clustering is not reflected in the phylogenetic analysis at the gene and protein level. In addition, the Acr3 proteins from *E. aurantiacum* PN47 and *E.* sp. S17 are structurally similar to the green cluster. Also, *E. mexicanum* HUD and *C. glutamicum* ATCC 13032 (BAB98863.1) Acr3 proteins are more structurally distant to the three clusters and do not possess the beta sheet.

Moreover, we found discontinuous membrane helices in the Acr3 modeled proteins belonging to the *Exiguobacterium* strains. Each structure contains a pair of discontinuous (an “α-helix – extended peptide – α-helix” motif) helix as described in Screpanti and Hunte (2007), which is associated with membrane proteins that transport ions in an active form. These discontinuous membrane helices are located between residues 93 and 119 (S4 Segment) and between residues 250 and 278 (S9 Segment; Supplementary Figure S2C). Both regions have conserved amino acids among the different Acr3 from *Exiguobacterium* strains studied, including a cysteine residue (position 107) of interest, which is conserved across the Acr3 family and has been described as essential for protein function (Fu et al., 2009). Furthermore, given the relevance of this residue, we searched for conformational interactions within a 5-angstroms range from the lateral chain of cysteine, and found that in all Acr3 proteins of the *Exiguobacterium* strains from SH and the reference S17 protein, the Cys-107 residue interacts with Thr-105, Pro-106, Thr-108, Asp-109, Trp-110, Tyr-111, Leu-129, Asn-132, Thr-260, Ala-263, Arg-264, Asn-265 and Glu-294. Nonetheless, there are some additional interactions: with Leu-136 in members from clusters II and III; with Ser-266 in members from clusters I and III; and with Pro-267 and Leu-298 exclusively in members from cluster III. Conversely, Glu-294 the other residue identified as key for Acr3 functions interacts with Leu-102, Cys-107, Trp-110, Phe-233, Leu-262, Ala-263, Arg-264, Ser-266, Ile-289, Gly-290, Pro-291, Leu-292, Ile-293, Leu-295, Pro-296, Val-297, Leu-298 and Val-299 in the Acr3 protein from all the studied strains. Interestingly, there are two more interaction with Tyr-111 and Leu-229 that only occur in cluster III members.

### Non-conserved amino acids

**Figure 5:**
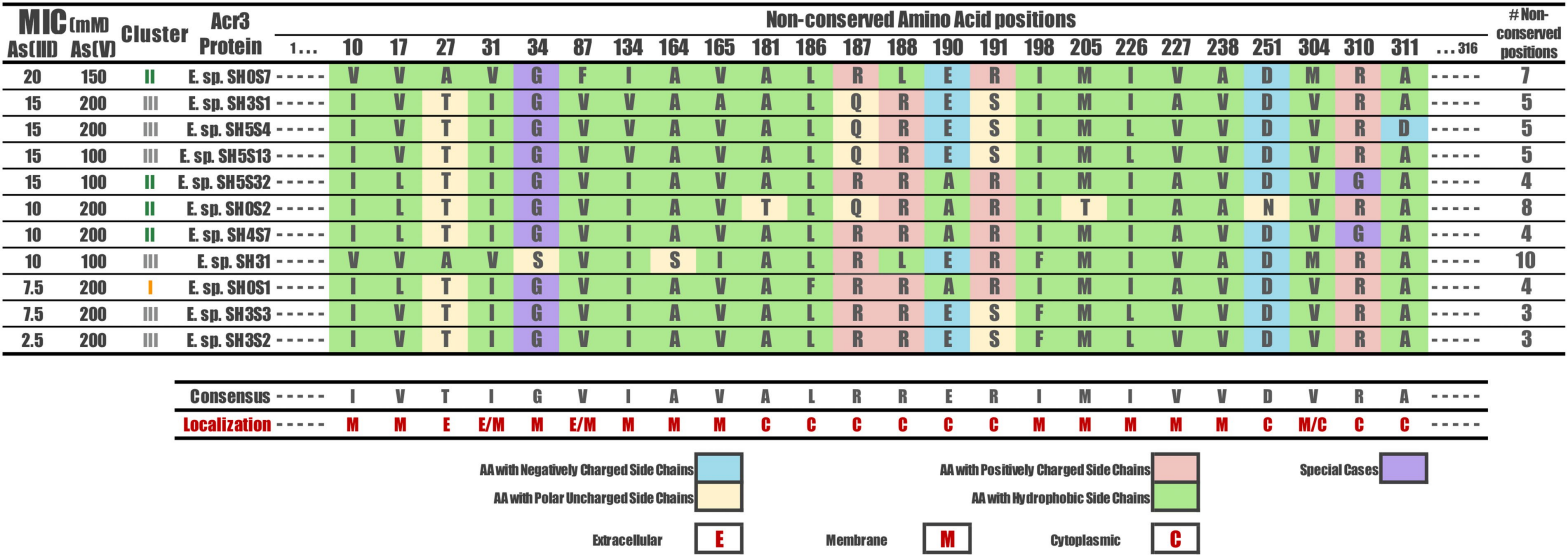
Non-conserved amino acids among the Acr3 protein sequences of *Exiguobacterium* strains. The amino acid occurring in each variable position identified is shown for every sequence, as well as the amino acidic classification according to the side chain and how many variable positions each one has, with respect to the consensus sequence. The topological location of each position variable is also shown (E, M, C). The strains are sorted according to their As(III) MIC value.

The non-conserved amino acids were identified by the alignment processes in their respective positions. These correspond to 24 amino acids across the *Exiguobacterium* strains Acr3 sequences, specifically at positions: 10, 17, 27, 31, 34, 87, 134, 164, 165, 181, 186, 187, 188, 190, 191, 198, 205, 226, 227, 238, 251, 304, 310 and 311 (Figure 5). The particular localization of each non-conserved amino acid in the three-dimensional protein can be seen in Figure 4B depicted as green spheres. Moreover, these non-conserved amino acids were identified and characterized according to the nature of their side chain. They correspond to amino acids with negatively-charged side chains (D, E), with positively-charged side chains (R, H, K), with polar-uncharged side chains (S, T, N, Q), with hydrophobic side chains (A, V, I, L, M, F, Y, W) and special cases (C, G, P). However, no correlation was found between the non-conserved amino acid positions and the resistance level presented by the strains to both As(III) and As(V). Even evaluating the type of mutation in each position, the nature of the amino acid, the number of non-conserved positions for each strain and differentiating between the three clusters, no association patterns were observed.

### Contact analysis

**Figure 6:**
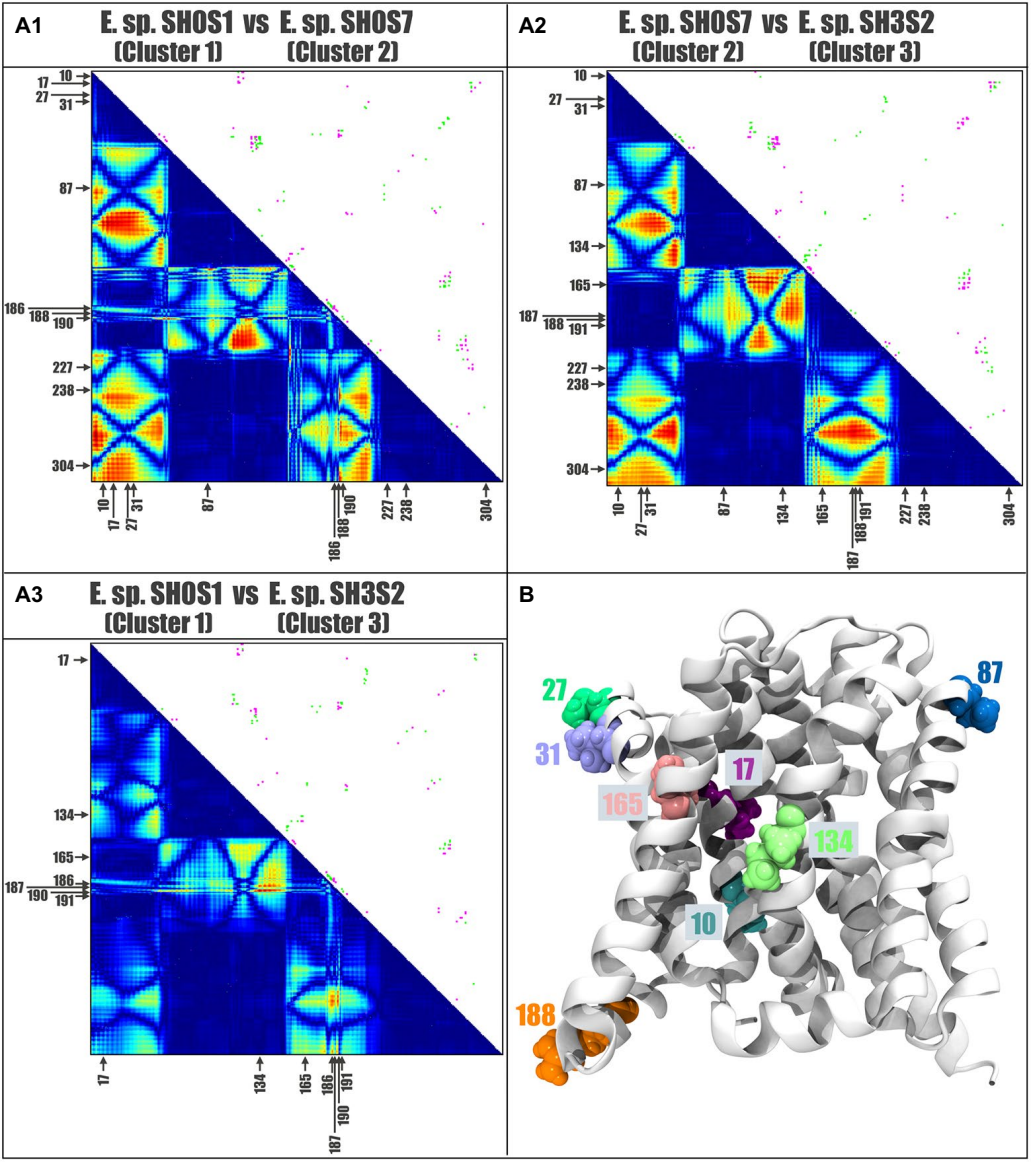
Contact analysis comparing selected Acr3 proteins from the *Exiguobacterium* strains. Contact maps: panels **(A1–A3)** shows the inter-cluster comparisons. The upper diagonal represents the pairwise comparison of the two contact maps: pink for the contacts that are unique to the first Acr3 protein of **A1**: *E.* sp. SH0S1, **A2**: *E.* sp. SH0S7, and **A3**: *E.* sp. SH0S1, and green the contacts unique to the second Acr3 protein of **A1**: *E.* sp. SH0S7, **A2**: *E.* sp. SH3S2, and **A3**: *E.* sp. SH3S2. The lower diagonal shows a heat-map of differences between the protein contacts, where red represents differences and blue represent similarities. The arrows indicate the positions of high variability and the amino acids for each case. **(B)** The position and localization of the eight residues with greatest variation are depicted by different colors on the structural model of the Acr3 protein.

A representative protein from each cluster was selected to measure the distance between all amino acid residue pairs of the protein structures using a two-dimensional matrix comparing two contact maps. This contact analysis was performed to determine the associated changes in the amino acid environment when a mutation occurs. For this analysis, Acr3 proteins from *E.* sp. SH0S1, *E.* sp. SH0S7 and *E.* sp. SH3S2 were selected, representing each of the three clusters (Figure 6A). Additionally, the Acr3 proteins from the *E.* sp. SH0S7 and *E.* sp. SH3S2 strains were found to be the most resistant to As(III) and the least resistant, respectively. We carried out an inter-cluster pair-wise comparison and observed that the Acr3 protein of *E.* sp. SH0S1 has a total of 1,575 contacts, while Acr3 of *E.* sp. SH0S7 has 1,576 contacts. The number of unique contacts for *E.* sp. SH0S1 is 82 and for *E.* sp. SH0S7 is 83. Also, the number of contacts in common between these proteins is 1,493 (Figure 6A1). Comparing the second case, Acr3 of *E.* sp. SH0S7 has a total of 1,576 contacts and *E.* sp. SH3S2 has a total of 1,577 contacts. The number of unique contacts for *E.* sp. SH0S7 is 81 and for *E.* sp. SH3S2 is 82. The number of contacts in common between these proteins is 1,495 (Figure 6A2). In the third comparison, Acr3 of *E.* sp. SH0S1 has a total of 1,575 contacts and *E.* sp. SH3S2 has a total of 1,577 contacts. The number of unique contacts for *E.* sp. SH0S7 is 61 and for *E.* sp. SH3S2 is 63. The number of contacts in common between these proteins is 1,514 (Figure 6A3). Although the number of total contacts is similar between the proteins, the unique contacts are more variable, especially in the third case. Also, the differences between the protein contacts indicate that the amino acids at postions 10, 17, 27, 31, 87, 134, 165 and 188 stand out (indicated by the arrows in Figure 6A), as they generate a difference in the interactions of the amino acids in their close environment that can be reflected by the structural differences of the generated models. Finally, Figure 6B shows the localization of these eight-most-relevant identified residues in the structural 3D model of Acr3 from *Exiguobacterium*. The binding energy results indicate that the amino acids 10 and 165 of cluster 1, represented by the *E*. sp. SH0S1 Acr3 protein, have a higher affinity (lower binding energy) for surrounding amino acids, as well as residue 134 for cluster 2 and residue 165 for cluster 3 (Supplementary Table S3). Therefore, these amino acids offer an increased stabilization of the protein. In addition, some variants such as leucine at position 17 have a higher affinity than valine, isoleucine at position 31 than valine, or leucine at position 188 than arginine. Likewise, it was observed that residue 87 does not present variations in its affinity, independent of the nature of the amino acid. The protein from SH0S7 (strain with greater As(III) resistance) has a differential pattern with respect to the other two strains analyzed, regarding the binding energy values of residues 31, 134, 165 and 188.

To delve into these differences, hydrogen bonds were identified and measured within a 5-angstrom radius of the amino acids of interest, namely, which varied in the contact analysis. We selected the eight previously identified variable residues (10, 17, 27, 31, 87, 134, 165 and 188) and the results can be seen in Table 3.

**Table 3:** Pairs of amino acids that form hydrogen bonds within 5 angstroms radius of residues with greatest variation.

Residue position	*E.* sp. SH0S1	*E.* sp. SH0S7	*E.* sp. SH3S2
10	Thr-18 – Leu-14 Leu-7 – Phe-4	Ser-15 – Phe-11 Leu-13 – Pro-9 Leu-14 – Val-10 Thr-18 – Leu-14	Phe-11 – Leu-7 Ser-15 – Phe-11 Ile-12 – Tyr-8 Leu-13 – Pro-9 Thr-18 – Leu-14
17	Thr-18 – Leu-14 Leu-20 – Val-16 Phe-22 – Thr-18 Thr-24 – Ala-21	Ser-15 – Phe-11 Leu-13 – Pro-9 Leu-20 – Val-16 Phe-22 – Thr-18 Thr-18 – Leu-14 Thr-24 – Ala-21	Ile-12 – Tyr-8 Leu-13 – Pro-9 Thr-18 – Leu-14 Ser-15 – Phe-11 Leu-20 – Val-16 Phe-22 – Thr-18 Thr-24 – Ala-21
27	Arg-30 – Glu-26 Ala-32 – Leu-28 Asp-33 – Arg-29	Ala-32 – Leu-28	Asp-33 – Arg-29
31	Arg-30 – Glu-26 Ala-32 – Leu-28 Asp-33 – Arg-29 Leu-40 – Ile-36	Ala-32 – Leu-28 Leu-40 – Ile-36	Asp-33 – Arg-29 Leu-40 – Ile-36
87	Ser-86 – Phe-82 Phe-82 – Pro-78 Phe-88 – Leu-84 Ala-85 – Ala-81	Ser-86 – Phe-82 Phe-82 – Pro-78 Leu-84 – Phe-80 Ala-85 – Ala-81 Trp-96 – His-92 Ile-100 – Trp-96	Phe-82 – Pro-78 Ser-86 – Phe-82 Leu-84 – Phe-80 Ala-85 – Ala-81
134	Leu-135 – Leu-131 Asn-132 – Ile-128 Gln-136 – Asn-132 Tyr-143 – Leu-139 Ile-174 – Leu-170	Leu-133 – Leu-129 Leu-135 – Leu-131 Gln-136 – Asn-132 Asn-132 – Ile-128 Tyr-143 – Leu-139 Ile-174 – Leu-170	Leu-133 – Leu-129 Leu-135 – Leu-131 Asn-132 – Ile-128 Gln-136 – Asn-132 Tyr-143 – Leu-139 Leu-163 – Phe-159 Ala-167 – Leu-163
165	Leu-160 – Val-156 Val-165 – His-161 His-161 – Ser-157 Ser-162 – Val-158 Ala-167 – Leu-163 Ser-172 – Ile-168 Thr-275 – Ala-271 Met-41 – Val-37 Leu-44 – Leu-40 Leu-40 – Ile-36 Phe-48 – Leu-44	His-161 – Ser-157 Ala-167 – Leu-163 Ile-168 – Ala-164 Gly-171 – Ala-167 Ser-172 – Ile-168 Thr-275 – Ala-271 Ala-276 – Ile-272 Thr-274 – Leu-270 Met-41 – Val-37 Leu-44 – Leu-40 Leu-40 – Ile-36 Phe-48 – Leu-44	Leu-160 – Val-156 Ala-165 – His-161 His-161 – Ser-157 Ser-162 – Val-158 Ile-168 – Ala-164 Ala-167 – Leu-163 Gly-171 – Ala-167 Ser-172 – Ile-168 Met-41 – Val-37 Phe-45 – Met-41 Leu-44 – Leu-40 Phe-48 – Leu-44
188	Arg-187 – Aan-184 Leu-189 – Gln-185 Ala-190 – Phe-186 Arg-191 – Arg-187	Leu-180 – Leu-176 Leu-188 – Gln-185 Ser-193 – Glu-190	Leu-180 – Leu-176 Ser-193 – Glu-190

**Figure 7:**
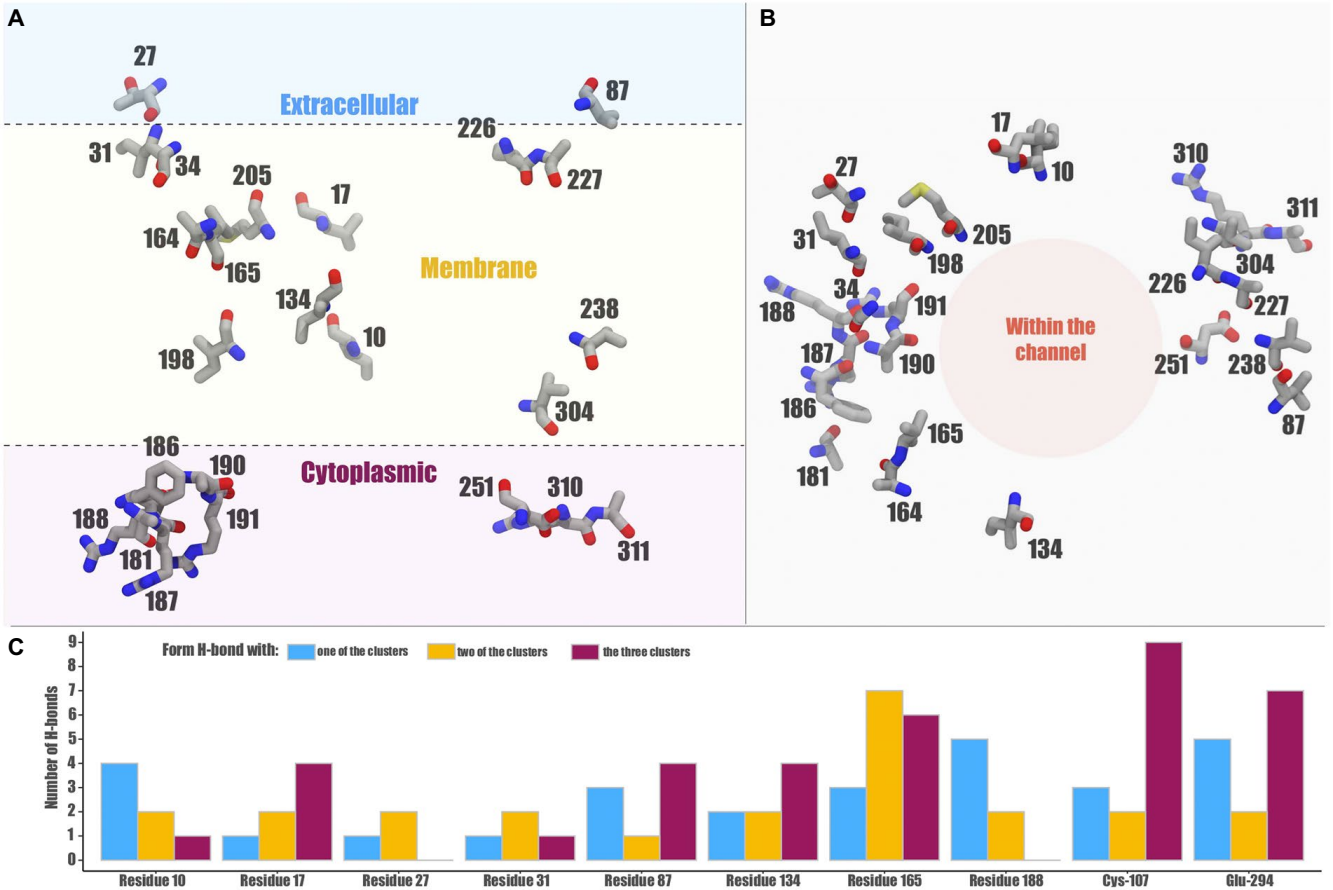
Distribution and possible effects of relevant residues in the *Exiguobacterium* Acr3 model. The location of non-conserved amino acids in the protein structure are shown **(A)** in a longitudinal view and **(B)** in a top-down view. Panel **(C)** shows the number of H-bonds that the non-conserved residues as well as the key Cys-107 and Glu-294 can form with proteins from the three clusters or exclusively with some of them.

By analyzing the distribution of the non-conserved amino acids, we found that many are located in the area where the alpha helices are in contact with the membrane lipids (away from the channel) and many are hydrophobic (Figures 7A,B). On the other hand, the amino acids located towards the cytoplasm are negatively-or positively-charged, and those within the extracellular region are uncharged polar or hydrophobic. However, by analyzing the hydrogen bonds formed by non-conserved or key residues, we found that indeed there are relevant changes, especially in those residues that form these bonds in some structures but not in others (Figure 7C). We highlight residues 27 and 188 that could be key in the loss or gain of interactions, since they are not present in all cases. Residue 10 could also be key, since its interaction is maintained in the 3 clusters, regardless of the mutation of this position and at the same time presents four single cluster exclusive interactions.

### Complemented bacteria arsenic minimal inhibitory concentration

The ability of the *Exiguobacterium* sp. SH31 (model strain) Acr3 pump to impact the arsenic resistance was tested through heterologous expression in susceptible *E. coli* strains. We determined the minimal inhibitory concentration (MIC) of each strain (Table 2) and found that as expected the complemented *E. coli* BW25113/pBR322-*acr3* strain has 2X and 5X increased resistance for As(III) and As(V) respectively, given that this strain possesses both the *ars* genes and the accessory *acr3* pump. Furthermore, the *E. coli* BW25113 strain with the *ars* genome (without the *acr3* pump) is 50% less resistant to As(III) and 37.5% less resistant to As(V) than the *E. coli* BW25113/pBR322-*acr3* strain. Moreover, the *E. coli* AW3110/pBR322-*acr3* strain (that does not harbor the *ars* operon but was complemented with the Acr3 pump) increased its ability to resist 2X more As(III) and 4X more As(V), compared to the complete sensitivity displayed by *E. coli* AW3110 (Table 4).

**Table 4:** Genotype of each strain used and their minimal inhibitory concentration against As(III) and As(V).

Strain	Genotype of interest	MIC (mM)	
		As(III)	As(V)
*E. coli* BW25113	*arsRCB* + / *acr3* −	1.25	15
*E. coli* BW25113/pBR322-*acr3*	*arsRCB* + / *acr3* +	2.5	40
*E. coli* AW3110	*arsRCB* − / *acr3* −	0.2	1.25
*E. coli* AW3110/ pBR322-*acr3*	*arsRCB* − / *acr3* +	1	5

### Growth monitoring

**Figure 8:**
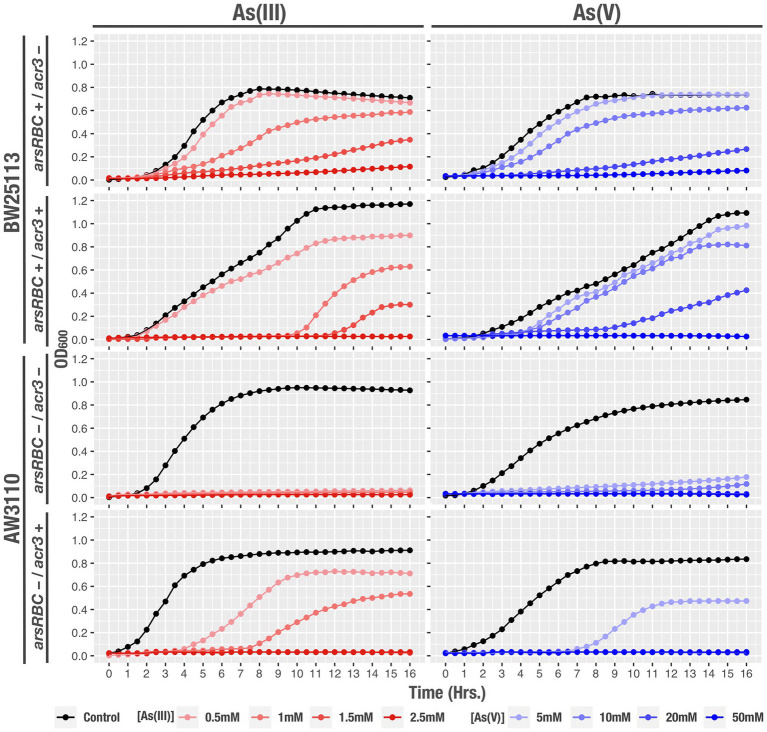
Growth curves of the studied strains, under the tested conditions: control, As(III) and As(V) at different concentrations. OD_600_ readings were recorded every hour during 16 h. Data represents an average of three independent experiments with three technical replicates each.

In order to further characterize the resistance of each strain to arsenic and determine the role of Acr3 in their ability to grow and develop in the presence of the toxic compound, we measured by spectrophotometry the bacterial growth curves at increasing concentrations of the metalloid. As expected, the strain with both the *ars* operon and the *acr3* gene had the ability to grow and reach exponential phase more quickly and efficiently than the other strains, despite the high As(III) and As(V) concentrations. *E. coli* BW25113 containing the classical resistance genes (*ars*), and the strain that only possessed *acr3* were able to reach exponential growth under conditions of 0.5 and 1 mM of As(III), and 5 mM of As(V), whereas the strain that lacked either mechanism did not grown in any of the conditions tested (Figure 8).

### Colony-forming unit determination

**Figure 9:**
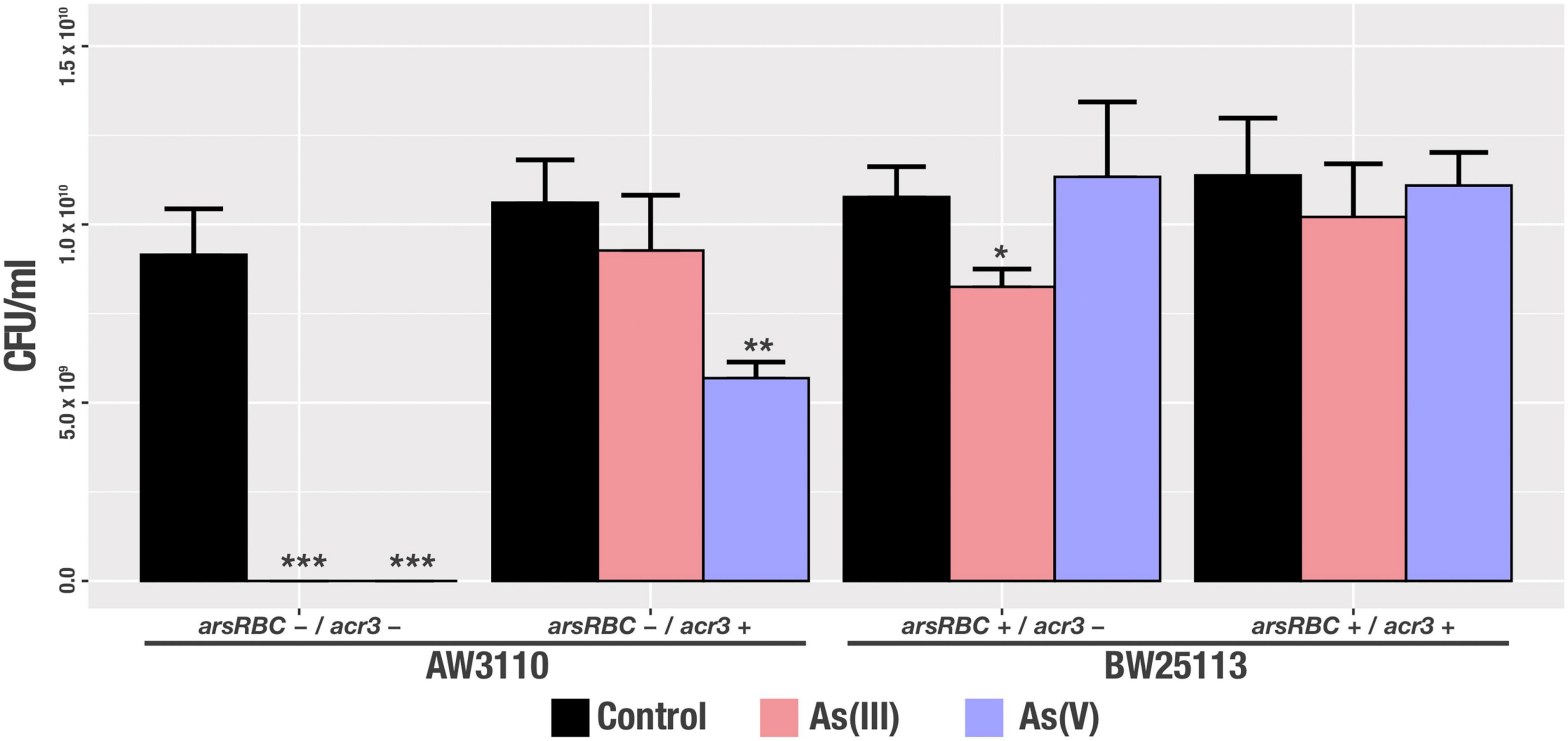
Viable cell recovery after arsenic treatment [control, As(III) and As(V)] of the studied strains. Bars represents an average of three independent experiments with three technical replicates each. * *p*<0.05; ** *p*<0.01; *** *p*<0.001.

Additionally, to determine the amount of viable bacteria that were able to survive the presence of arsenic, we measured the Colony Forming Units (CFU) that were generated after growth in media containing As(III) and As(V) for the same strains. Our results show statistically significant differences in cell viability with the strains AW3110 *arsRBC−/acr3+* after As(III) exposure and BW25113 *arsRBC+/acr3-in* the presence of As(III) compared to the control condition. Bacteria that lacked the *ars* operon (AW3110 *arsRBC-acr3+*) had significantly decreased cell viability in the presence of As(V) and the strains without *acr3* (BW25113 *ars + acr3-*) had lower recovery under As(III) treatment (Figure 9).

## Discussion

The prevalence of arsenite transporters in contaminated and non-contaminated soils has always attracted research. Acr3 transporter sequences are more common than those of ArsB, and have been found across every phylogenetic domain conferring resistance to As(III) (Achour et al., 2007; Yang et al., 2012). This is also reflected by a wider distribution of Acr3 in sequenced bacterial genomes (Shi et al., 2018). The evolution of several means of arsenic detoxification is the result of its environmental prevalence. Arsenic efflux is the most common detoxification mechanism and a considerable diversity of transporter families has been described (Ghosh et al., 1999; Yang et al., 2015). Our group came to the same conclusion, by evaluating at the metagenomic level the abundance and distribution of arsenic efflux pumps in the arsenic rich sediment from SH (Castro-Severyn et al., 2021). ArsB is an antiporter [exchanging As(III) for H], where counter-transport is driven by the protonmotive force, and some experiments have indicated that arsenite efflux *via* Acr3 could also be coupled to protonmotive force (Ghosh et al., 1999; Villadangos et al., 2012). On the other hand, there is also evidence that demostrate that Acr3 is a uniporter, with As(III) efflux coupled to the membrane potential (Fu et al., 2009). Therefore, despite the widespread distribution of Acr3, its transport mechanism remains unclear.

To identify the genomic basis of these phenotypic capabilities, we addressed the genomic context, phylogenic relationships, as well as protein structure and amino acidic interactions, further elucidating the underlying mechanisms that influence the functionality of Acr3 and its contribution to the variety of resistance levels observed among the studied *Exiguobacterium* strains. Hence, the phylogeny of the *acr3* gene sequences show a clear grouping of all strains from the SH, indicating their close evolutionary history. This could be attributed to the isolation, uniqueness, and diversity of the ecosystem, where bacterial communities change and adapt, but basic functions, such as resistance to metal(oids) is critical given the geochemical characteristics of the area (Risacher et al., 2003; Dorador et al., 2013; Hernández et al., 2016). Therefore, the results of the dN/dS ratio indicate that the *acr3* genes of these bacteria are under negative selection, that could be attributed to the mechanism in which the selective forces tend to maintain an improved structure/function, thus contributing to the stability of the trait without generating further mutations. The dN/dS ratio remains one of the most popular and reliable measures of evolutionary pressures on protein-coding regions, with a simple and intuitive interpretation of dN/dS < 1 as negative selection, dN/dS = 1 as neutrality, and dN/dS > 1 as positive selection (Kryazhimskiy and Plotkin, 2008).

Moreover, the study of the genetic context of the *acr3* gene could shed light on associated functions, evolution and regulatory networks. Among the *Exiguobacterium* strains from SH we found two distinct versions of the gene cluster, differing mainly due to the presence of one small hypothetical protein, with an unknown function but belonging to a cysteine-rich domain superfamily that could be important for arsenic resistance; this protein thus requires further biochemical and functional studies. However, it is relevant to highlight that strains S17 and PN47, used as references, are closely related to the ones isolated from the SH, as they belong to nearby places in the Altiplano-Puna Region, where arsenic concentrations are also high (Ordoñez et al., 2013; Strahsburger et al., 2018; Castro-Severyn et al., 2020). This differs from the situation of the HUD strain, which is totally different and whose arsenic resistance has not been experimentally demonstrated.

To study a transport protein at the functional level, it is vital to have a basic understanding of its structure. Hence, the first ever experimentally derived structural study of the Acr3 family concluded that the Acr3 protein from *Bacillus subtilis* has 10 transmembrane helices, short loops connecting the membrane spanning segments and cytoplasmic loops longer than the extracellular ones (Aaltonen and Silow, 2008). Other Acr3 proteins from organisms such as *C. glutamicum, Exiguobacterium* sp.*, S. cerevisiae* and *A. metalliredigens* share the same protein structure (Maciaszczyk-Dziubinska et al., 2012; Ordoñez et al., 2015; Hwang et al., 2016; Mateos et al., 2017). Our findings of predicted topology agree with those previous studies indicating that *acr3* encodes a transport protein with transmembrane characteristics (ten transmembrane segments), and structural similarities between the sequences studied also show the formation of three distinct groups. Additionally, we determined that the discontinuous alpha-helices are in the same area as the conserved residue Cys-107, which is distinctive of the Sodium/Bile Acid symporter family (Ordoñez et al., 2015).

However, the grouping of the proteins in three clusters does not reflect the phylogenetic relationships. Therefore, these differences are associated with structural traits that could influence the efficiency of the protein. For instance, the protein crystal used as reference has a beta-sheet that was not present in the *Exiguobacterium* Acr3 proteins; this common motif could modify the interaction with the membrane or the accessibility to the extracellular space. Nonetheless, our predicted topology is consistent with previous proposals for *B. subtilis* and *A. metalliredigens* (Aaltonen and Silow, 2008; Fu et al., 2009).

The Acr3 from *S. cerevisiae* is the best characterized member of the family, conferring resistance to high concentrations of As(III) and cells lacking this protein are hypersensitive and accumulate more As(III) compared to wild type. A complete loss of transport activity was evident in mutant cells in which the highly conserved cysteine residue was eliminated (Cys151Ala; Maciaszczyk-Dziubinska et al., 2012). The same conclusion was reached in another study where a modification of the same cysteine residue that is conserved in all analyzed Acr3 homologues resulted in loss of transport activity (Fu et al., 2009). This residue is the only cysteine in the Acr3 protein. It is predicted to be in the fourth transmembrane segment (a hydrophobic region), located right in the middle of a clear channel in the structure, and is proposed to be the catalytic residue that binds arsenite (Aaltonen and Silow, 2008), which strongly suggests that the thiol group is required to interact with the As(III) for translocation and thus activation of transport. Also, as single thiols bind trivalent arsenic with low affinity, the binding to As(III) might be weak on one side of the membrane, so the low affinity would facilitate release of As(III) from the carrier on the other side of the membrane (Fu et al., 2009).

The Acr3 proteins of the studied *Exiguobacterium* strains and S17, share the presence of an intracellular region with a preponderance of positively charged residues (Arg-3, Arg-57, Arg-63, Arg-177, Arg-191, Arg-241, Arg-242, Arg-249, Lys-6, Lys-119, Lys-182, Lys-245, Lys-246), compared with the number of negative residues (Asp-121, Asp-251, Glu-5, Glu-190). Meanwhile, on the extracellular side, there are more negative residues (Asp-33, Asp-91, Glu-26, Glu-217, Glu-250, Glu-280) than positive ones (Arg-29, Arg-30, Arg-213). As the pKa of arsenite is 9.2 and the *Exiguobacterium* genus is known to be alkaliphile, a low percentage of arsenite would be ionized on the cytosolic side of the membrane, where positively charged residues would interact electrostatically with arsenite due to its negative charge. Interestingly, there are three charged residues (Arg-264, Asp-109, Glu-294) in the core of the transmembrane segments, which are close to Cys-107; all four of them are placed in the catalytic area, and so could be involved in arsenite binding (Ordoñez et al., 2015).

Although the relationship between individual residues with the function of Acr3 proteins is not completely clear, the binding of As(III) by cysteine residues is common for a number of arsenic resistance proteins, including the ArsR repressor, the ArsD metallochaperone, and the ArsA ATPase (Villadangos et al., 2012). In addition, another residue that has been proposed as possibly having great relevance in proton translocation during As(III)/H+ antiport by *C. glutamicum* Acr3 is Glu-305, whose mutation also causes loss of transport capacity (Maciaszczyk-Dziubinska et al., 2012; Villadangos et al., 2012). The involvement of glutamic acid in proton translocation has been previously described for proteins such as cytochromes and haem-copper oxidases (Riistama et al., 1997; Verkhovskaya et al., 1997; Backgren et al., 2000). Only substitutions of Acr3 Cys and Glu altered As(III) resistance in homologous (*C. glutamicum*) and heterologous (*E. coli*) expression analyses, supporting the key role for these two amino acids in As(III) translocation and cellular release (Mateos et al., 2017). Also, as both amino acids are located in the middle of transmembrane segments it has been speculated that both could serve as selectivity filters for As(III).

In this context we evaluated the interaction between these key amino acids (Cys-107 and Glu-294; part of the active site of the protein) and with others in each strain, finding that Cys-107 can interact with a total of 17 residues in the 3D structure, of which 13 interactions occur in all the studied strains, leaving 4 interactions exclusive to some strains. On the other hand, Glu-294 can interact with a total of 20 amino acids in the 3D structure, of which 18 interactions occur in all the studied strains whilst only 2 are exclusive to some strains. All amino acids that are part of these interactions are fully conserved in all of the Acr3 proteins studied; thus the differences or the inability to form some interactions in some sequences may be due to the influence exerted on the 3D structure by the variation level of 24 amino acids positions that are not conserved in each particular sequence.

There is a potential impact of conserved amino acids over Acr3 functionality. As this level of conservation is observed in our studied proteins, we aimed to elucidate the role or participation of variable amino acids in the different arsenic resistance levels observed among the SH *Exiguobacterium* strains. We found variation within the proteins based on non-conserved amino acids by identifying changes and their position. Despite not finding any association patterns between the non-conserved positions and the arsenic resistance level of each strain, we found that the variations are associated with the more flexible loops; but not within the channel nor the motifs that interact with the membrane. Moreover, the amino acids that vary in the extracellular region are uncharged polar or hydrophobic, while the residues that change in the membrane are hydrophobic as well as those residues found in the helices that are in contact with the membrane lipids on the outside of the channel. However, the amino acids that are located towards the cytoplasm are negatively-or positively-charged, possibly forming a charged channel. Finally, the conservation or variation of this composition directly influences the critical electrostatic interactions of the influx/efflux of ions, as can be seen in Figure 7.

Considering the level of variation observed between the sequences (24 of the 316 positions), we aimed to evaluate if these changes could derive into modifications in interactions or contacts, thus impacting the protein structure or function. We found that residues 27 and 31, located in the predicted membrane/extracellular interface are key as they could be important for the entry of ions into the cell, as well as residue 186 located in the part of the cytoplasm/membrane interface that is vertical to residues 27 and 31, thus possibly affecting the entry or exit of arsenic. Also, it is well known that hydrogen bonds stabilize alpha-helices (Senes et al., 2001; Jarosch, 2005). In the case of Acr3, we detected important changes in these interactions between the different clusters, mainly due to non-conserved amino acids, which clearly exert an effect on the pump structure and dynamics, that could derive into functional differences. Finally, the analysis of the contact maps shows that interactions between clusters 1 and 3 behave similarly, as there are minimal hot-spots that are only found in the charged amino acids of the cytoplasmic region. However, comparing the contact maps of cluster 1 vs. cluster 2 and cluster 2 vs. cluster 3, we detected major differences. There appears to be a pattern where amino acids from intra or inter cellular regions are key for pump function, as they modify the contacts around them, whilst conservative substitutions of hydrophobic amino acids in the transmembrane regions, do not seem to alter the surroundings. Measurement of binding energy shows a distinctive pattern in some Acr3 residues for the most As(III) resistant strain; however these possible relationships need to be experimentally demonstrated.

Finally, the acquisition of the Acr3 pump increases the organism’s tolerance to arsenic exposure, as has been shown before (Ali et al., 2012; Chen et al., 2017; Yang et al., 2022). Of particular relevance is the successful cloning and heterologous expression of a proteorhodopsin gene from a halotolerant, UV-and arsenic-resistant *Exiguobacterium* sp. S17 isolated from a high-altitude Andean Lake in *E. coli* XL1 (Albarracín et al., 2016). Our results with the heterologous expression of *acr3* indicate that, as expected, the presence of the gene that encodes for the Acr3 pump increase the ability of the strains to withstand greater concentrations of arsenite and arsenate, by enhancing resistance to arsenic (Table 4), growth capability (Figure 8) and cell viability (Figure 9) when propagated in high concentrations of the toxic compounds.

## Conclusion

Our results indicate that the arsenic pump Acr3 plays an active role in the ability to detoxify the cell and resist higher concentrations of arsenic in bacteria under laboratory-controlled conditions. Heterologous acquisition of Arc3 provides augmented resistance to this toxic compound. At the genetic level the *acr3* sequences from the SH strains have a close evolutionary history, which can be influenced by environmental conditions. Genetic cluster analyses show that one hypothetical protein with an unknown function marks the difference within the genetic context of the *acr3* gene. At the protein structural level, observed differences segregate the pumps into three different clusters, where the non-conserved amino acids could impact the function of the protein. Consequently, we are able to observe changes in many interactions such as 5-angstrom contacts and hydrogen bonds. Indeed, the Cys-107 and Glu-294 residues seem to be in critical positions, and are key for the functionality. In any case, further molecular dynamics and structural studies are necessary to elucidate whether these interactions and changes within the active site are relevant for protein efficiency and the ability of the strain to eliminate arsenic.

## Data availability statement

Publicly available datasets were analyzed in this study. This data can be found at: DDBJ/ENA/GenBank under the Bioproject: PRJNA319980.

## Author contributions

JC-S and CS conceived and designed the study. JC-S and VG performed all experimental procedures. JC-S and IA-D performed all bioinformatic analyzes. JC-S, IA-D, and CP-E analyzed interpreted all data and wrote the first manuscript draft. CS and FR contributed with reagents, materials, and analysis. All authors read and approved the final manuscript.

## Funding

This research was sponsored by ANID (Agencia Nacional de Investigación y Desarrollo de Chile) grants. CS was funded by ANID-FONDECYT Regular 1210633 and ECOS-ANID 170023. JC-S was funded by ANID 2021 Post-Doctoral FONDECYT 3210156. FR was funded by ANID-FONDECYT Regular 1220902. The funders had no role in study design, data collection and analysis, decision to publish, or preparation of the manuscript.

## Conflict of interest

The authors declare that the research was conducted in the absence of any commercial or financial relationships that could be construed as a potential conflict of interest.

## Publisher’s note

All claims expressed in this article are solely those of the authors and do not necessarily represent those of their affiliated organizations, or those of the publisher, the editors and the reviewers. Any product that may be evaluated in this article, or claim that may be made by its manufacturer, is not guaranteed or endorsed by the publisher.
