# Supplementary material for: Biochemical, genomic and structural characteristics of the Acr3 pump in Exiguobacterium strains isolated from arsenic-rich Salar de Huasco sediments
Source: Front Microbiol. 2022 Nov 3;13:1047283. doi: 10.3389/fmicb.2022.1047283 (PMC9671657; doi:10.3389/fmicb.2022.1047283)
Supplement: Supplementary file 1 [file Data_Sheet_1.PDF]

SUPPLEMENTARY MATERIAL

Supplementary Table S1. Indentity and dN/dS ratios of the studied *acr3* sequences, with respect to the S17 sequence (used as reference).

Strain	S	N	dS	dN	dN/dS ratio	Identity
**SH0S1**	281.6	666.4	0.1951	0.0266	0.1366	93.5%
**SH0S2**	278.8	669.2	0.1910	0.0266	0.1391	93.6%
**SH0S7**	294.4	653.6	0.2062	0.0289	0.1403	93.0%
**SH3S1**	281.6	666.4	0.1634	0.0216	0.1321	94.4%
**SH3S2**	289.9	658.1	0.2108	0.0253	0.1202	93.2%
**SH3S3**	289.9	658.1	0.2108	0.0253	0.1202	93.2%
**SH31**	297.3	650.7	0.2210	0.0306	0.1386	92.5%
**SH4S7**	280.3	667.7	0.1950	0.0266	0.1364	93.5%
**SH5S4**	281.2	666.8	0.1728	0.0231	0.1339	94.2%
**SH5S13**	280.3	667.7	0.1950	0.0266	0.1364	94.1%
**SH5S32**	283.2	664.8	0.1730	0.0216	0.1251	93.5%

S: Number of synonymous sites, N: Number of non-synonymous sites, dS: Synonymous substitution rate, dN: Non-Synonymous substitution rate.

Supplementary Table S2. The RMSD distance measurement between alpha carbons were calculated for all proteins against the template (crystal structure of the bile acid sodium transporter of *Neisseria meningitidis*; PDB: 3ZUX_A).

Acr3 protein	RMSD (Å)
Alkaliphilus metalliredigens QYMF (ABR47201.1)	23.3
Alkaliphilus metalliredigens QYMF (ABR47998.1)	23.7
Alkaliphilus metalliredigens QYMF (ABR50078.1)	23.5
Corynebacterium glutamicum ATCC 13032 (BAB97655.1)	22.5
Corynebacterium glutamicum ATCC 13032 (BAB98863.1)	10.17
Corynebacterium glutamicum ATCC 13032 (BAB98903.1)	23.8
Bacillus subtilis 168	22.3
Exiguobacterium mexicanum HUD	7.6
Exiguobacterium sp. S17	6.4
Exiguobacterium. sp. SH0S1	5.6
Exiguobacterium. sp. SH0S2	5.6
Exiguobacterium. sp. SH0S7	5.7
Exiguobacterium. sp. SH3S1	6.5
Exiguobacterium. sp. SH3S2	6.5
Exiguobacterium. sp. SH3S3	6.5
Exiguobacterium. sp. SH4S7	6.0
Exiguobacterium. sp. SH5S4	6.4
Exiguobacterium. sp. SH5S13	6.5
Exiguobacterium. sp. SH5S32	6.0
Exiguobacterium. sp. SH31	6.5

Supplementary Table S3. Binding energy of the amino acids with greater variations, and residues within 5 angstroms radius of the amino acid. The amino acids under study are shown in bold. In parentheses are the residues of each protein by position. All values are in kcal/mol.

Residue	*E.* sp. SH0S1	*E.* sp. SH0S7	*E.* sp. SH3S2
**10**	-23.89 (I)	-11.82 (V)	-13.22 (I)
**17**	-15.48 (L)	-12.8 (V)	-12.3 (V)
**27**	-8.22 (T)	-9.08 (A)	-12.44 (T)
**31**	-16.7 (I)	-11.85 (V)	-16.59 (I)
**87**	-12.01 (V)	-12.75 (F)	-12.26 (V)
**134**	-17.1 (I)	-22.59 (I)	-16.63 (I)
**165**	-26.82 (V)	-7.36 (V)	-23.57 (V)
**188**	-4.29 (R)	-16.47 (L)	-3.63 (R)

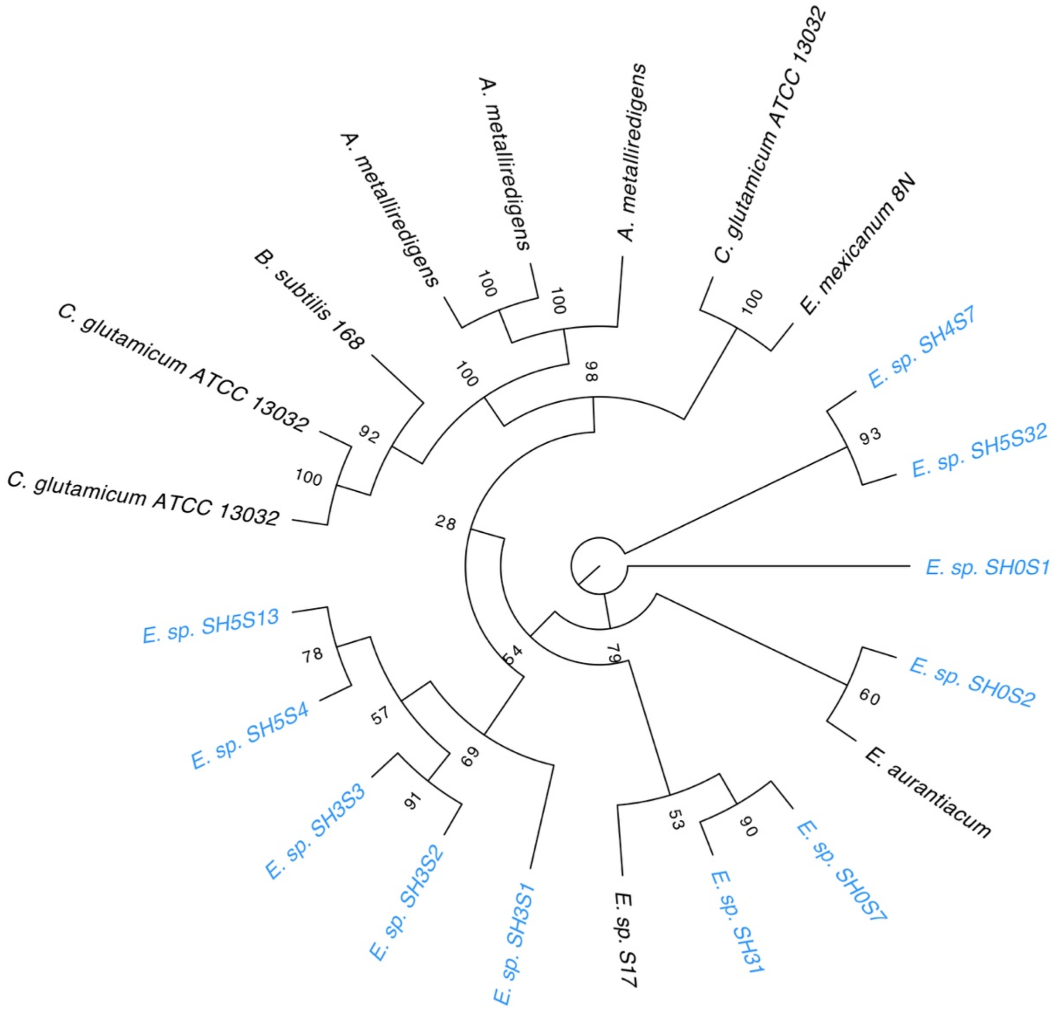

Supplementary Figure S1. Maximum-likelihood phylogeny reconstruction with all Acr3 aminoacidic sequences compared (*Exiguobacterium* strains and references).

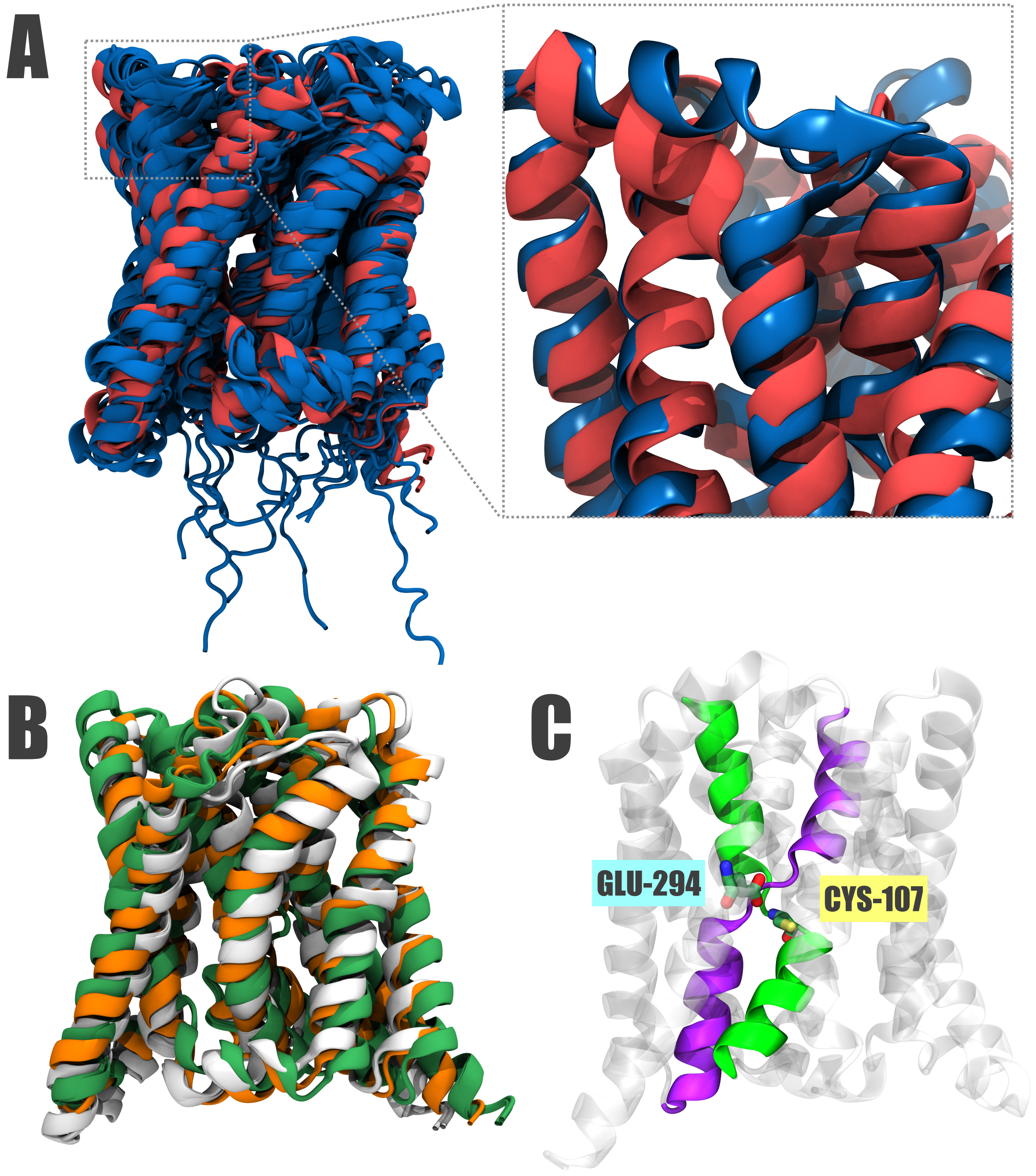

Supplementary Figure S2. Acr3 protein structural characteristics. A) *Exiguobacterium* models (in blue) and references models (in red), inset is showing the presence of the extra beta-sheet in the references models. B) Structural superposition of the 3D models from the three different clusters (cluster I in orange, cluster II in green and cluster III in gray). C) Representation of the two discontinuous alpha helices and the conserved Cys-107 and Glu-294 residues localization.
